# The Production and Utilization of GDP-glucose in the Biosynthesis of Trehalose 6-Phosphate by Streptomyces venezuelae*

**DOI:** 10.1074/jbc.M116.758664

**Published:** 2016-11-30

**Authors:** Matías D. Asención Diez, Farzana Miah, Clare E. M. Stevenson, David M. Lawson, Alberto A. Iglesias, Stephen Bornemann

**Affiliations:** From the § Biological Chemistry Department, John Innes Centre, Norwich Research Park, Norwich NR4 7UH, United Kingdom and; the ‡ Laboratorio de Enzimología Molecular, Instituto de Agrobiotecnología del Litoral (UNL-CONICET), Facultad de Bioquímica y Ciencias Biológicas, CCT-Santa Fe, Colectora Ruta Nac 168 Km 0, 3000 Santa Fe, Argentina

**Keywords:** carbohydrate metabolism, crystal structure, glycosyltransferase, microbiology, substrate specificity, trehalose

## Abstract

Trehalose-6-phosphate synthase OtsA from streptomycetes is unusual in that it uses GDP-glucose as the donor substrate rather than the more commonly used UDP-glucose. We now confirm that OtsA from *Streptomyces venezuelae* has such a preference for GDP-glucose and can utilize ADP-glucose to some extent too. A crystal structure of the enzyme shows that it shares twin Rossmann-like domains with the UDP-glucose-specific OtsA from *Escherichia coli*. However, it is structurally more similar to *Streptomyces hygroscopicus* VldE, a GDP-valienol-dependent pseudoglycosyltransferase enzyme. Comparison of the donor binding sites reveals that the amino acids associated with the binding of diphosphoribose are almost all identical in these three enzymes. By contrast, the amino acids associated with binding guanine in VldE (Asn, Thr, and Val) are similar in *S. venezuelae* OtsA (Asp, Ser, and Phe, respectively) but not conserved in *E. coli* OtsA (His, Leu, and Asp, respectively), providing a rationale for the purine base specificity of *S. venezuelae* OtsA. To establish which donor is used *in vivo*, we generated an *otsA* null mutant in *S. venezuelae*. The mutant had a cell density-dependent growth phenotype and accumulated galactose 1-phosphate, glucose 1-phosphate, and GDP-glucose when grown on galactose. To determine how the GDP-glucose is generated, we characterized three candidate GDP-glucose pyrophosphorylases. SVEN_3027 is a UDP-glucose pyrophosphorylase, SVEN_3972 is an unusual ITP-mannose pyrophosphorylase, and SVEN_2781 is a pyrophosphorylase that is capable of generating GDP-glucose as well as GDP-mannose. We have therefore established how *S. venezuelae* can make and utilize GDP-glucose in the biosynthesis of trehalose 6-phosphate.

## Introduction

**FIGURE 1.**
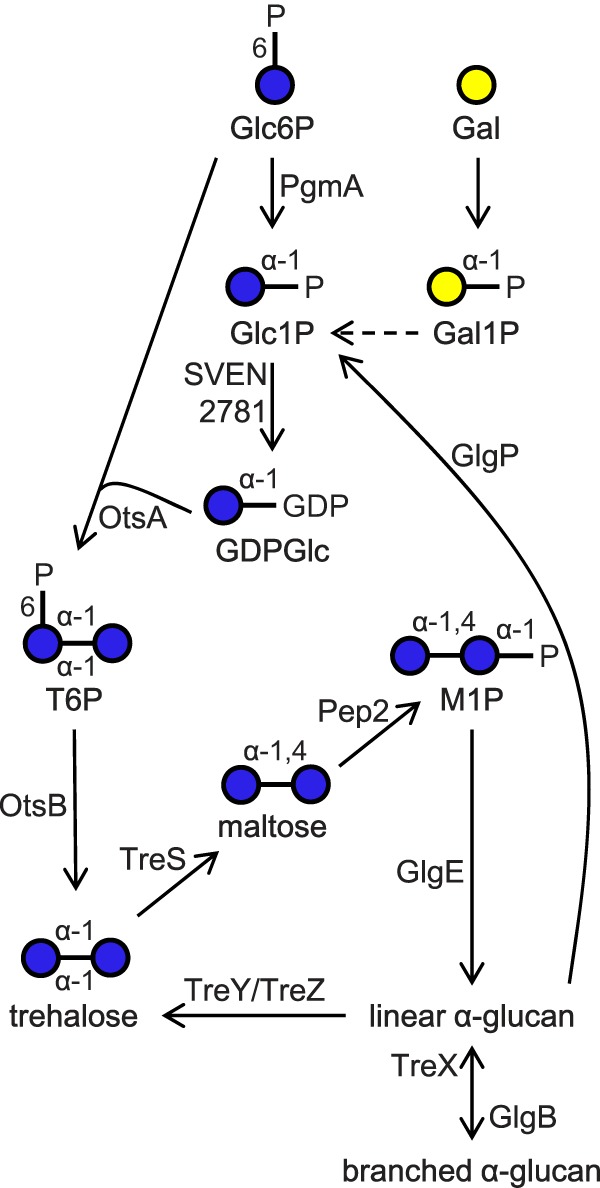
**Proposed metabolic pathways connecting galactose with GDP-glucose.** The ability of SVEN_2781 to produce GDP-glucose was established in this study. The conversion of galactose 1-phosphate to glucose 1-phosphate probably occurs via the epimerization of an NDP-galactose in a Leloir-type pathway (31). *Glc6P*, glucose 6-phosphate; *Glc1P*, glucose 1-phosphate; *Gal*, galactose; *Gal1P*, galactose 1-phosphate; *GDPGlc*, GDP-glucose; *T6P*, trehalose 6-phosphate; *M1P*, maltose 1-phosphate.

*Streptomyces venezuelae* is a soil-dwelling bacterium with a developmental life cycle that initiates with the germination of spores (1). Vegetative hyphae then form to generate a substrate mycelium. Finally, aerial hyphae form that differentiate into the next generation of spores. The spores contain trehalose as a carbon and energy source for germination (2). This non-reducing disaccharide (α-d-glucopyranosyl-(1→1)-α-d-glucopyranoside) is also known to provide tolerance to stresses such as desiccation, dehydration, heat, cold, and oxidation (3). In addition, trehalose is utilized by the GlgE pathway (4–7) in this organism (8) for the transient biosynthesis of a glycogen-like α-glucan (Fig. 1) (9). This polymer is disassembled in streptomycetes by TreY (EC 5.4.99.15, (1→4)-α-d-glucan 1-α-d-glucosylmutase) and TreZ (EC 3.2.1.141, 4-α-d-(1,4-α-d-glucano)trehalose glucanohydrolase (trehalose-producing)) to regenerate trehalose during the onset of sporulation (10–14).

The only route for the *de novo* biosynthesis of trehalose in *S. venezuelae* is via trehalose 6-phosphate (8) (Fig. 1). OtsA (α,α-trehalose-phosphate synthase) is responsible for the formation of this metabolic intermediate from an NDP-glucose donor and glucose 6-phosphate as the acceptor. The enzyme from *Streptomyces hygroscopicus* and some other actinomycetes has been reported to exhibit a preference for GDP-glucose as the donor (EC 2.4.1.36, GDP-glucose:d-glucose-6-phosphate 1-α-d-glucosyltransferase, of the GT20 CAZy family) (15–19). This contrasts with OtsA enzymes from other bacteria, insects, yeasts, and fungi that most commonly utilize UDP-glucose as the donor substrate (EC 2.4.1.15, UDP-glucose:d-glucose-6-phosphate 1-α-d-glucosyltransferase). For example, the enzyme from *Escherichia coli* is UDP-glucose-specific, with crystal structures providing a clear understanding of the structural basis for its donor preference (20, 21). In all cases, trehalose 6-phosphate is dephosphorylated by OtsB (EC 3.1.3.12, trehalose-6-phosphate phosphohydrolase) to give trehalose.

Because OtsA enzymes in streptomycetes use GDP-glucose as the donor, it would be expected that these organisms possess a GDP-glucose pyrophosphorylase (EC 2.7.7.34, GTP:α-d-glucose-1-phosphate guanylyltransferase) capable of forming GDP-glucose from GTP and glucose 1-phosphate. Such enzyme activity has been reported in mammalian cells, plant tissues, and streptomycetes (22–26), but no sequence information is available, and no bacterial enzyme has been characterized to date. We therefore determined the donor preference of OtsA from *S. venezuelae* both *in vitro* and *in vivo*, identified its structural basis, and characterized three candidate enzymes for the production of the preferred donor.

## Results

### 

#### 

##### GDP-glucose Is the Preferred Donor Substrate of Recombinant S. venezuelae OtsA

The enzyme OtsA from *S. hygroscopicus* and other streptomycetes has been reported to have a preference for the donor GDP-glucose (15–17). To establish whether the enzyme from *S. venezuelae* shares this donor preference, the recombinant enzyme was produced in *E. coli*. The theoretical size of the protein with its tag is 51 kDa, but the recombinant protein had an estimated molecular mass of 109 kDa according to size exclusion chromatography, consistent with it forming a homodimer in solution.

By monitoring the generation of nucleotide sugar diphosphate in a coupled assay, it was clear that the affinity of the enzyme for glucose 6-phosphate was independent of the GDP-glucose or ADP-glucose donor substrate used (Table 1). However, the associated value of *k*_cat_ was nearly 5-fold greater with GDP-glucose. Furthermore, the *k*_cat_ and *K_m_* for GDP-glucose were more favorable than with ADP-glucose, giving a catalytic efficiency an order of magnitude greater (Table 1). No activity with UDP-glucose, UDP-galactose, or GDP-mannose was detected. In addition, none of these three compounds inhibited enzyme activity when used at the same concentration as either GDP-glucose or ADP-glucose, implying that they do not bind to the enzyme active site. The preference for the donor substrates was confirmed using ^1^H NMR spectroscopy to monitor the reactions. Potential allosteric regulators of OtsA were tested (fructose 6-phosphate, glucose 1-phosphate, mannose 1-phosphate, GTP, ATP, pyrophosphate, and orthophosphate), but none showed any effect on enzyme activity with GDP-glucose. This contrasts with the activation of the *Mycobacterium tuberculosis* enzyme by fructose 6-phosphate (27). Therefore, although able to use another purine diphosphoglucose donor, the enzyme had a preference for GDP-glucose and was not subject to allosteric regulation.

**TABLE 1:** **Kinetic analysis of recombinant *S. venezuelae* OtsA**

Substrate	Fixed substrate (concentration)	*k*_cat_	*K_m_*	*k*_cat_/*K_m_*
		*s*^−*1*^	*mm*	*mm*^−*1*^ *s*^−*1*^
GDP-glucose	Glucose 6-phosphate (5 mm)	139 ± 8	0.12 ± 0.02	1143 ± 223
Glucose 6-phosphate	GDP-glucose (0.5 mm)	123 ± 3	0.93 ± 0.06	132 ± 9
ADP-glucose	Glucose 6-phosphate (5 mm)	29 ± 1	0.44 ± 0.05	67 ± 7
Glucose 6-phosphate	ADP-glucose (2.5 mm)	26 ± 1	0.90 ± 0.07	29 ± 2

##### The Structural Basis for Donor Specificity

To establish the structural basis for the donor specificity of *S. venezuelae* OtsA, the recombinant enzyme was crystallized. Crystals diffracted to 1.95 Å (Table 2), allowing the protein structure to be solved using molecular replacement. This was done using a Phyre^2^-generated homology model (28) for OtsA based on the structure of *S. hygroscopicus* VldE (PDB^3^ code 3T5T (29)), a protein of known structure with which it shares one of the highest sequence identities (30%). VldE catalyzes the formation of validoxylamine A 7′-phosphate with a non-glycosidic C–N bond from GDP-valienol and validamine 7-phosphate.

**TABLE 2:** **Summary of X-ray data and model parameters for *S. venezuelae* OtsA** Values in parentheses are for the outer resolution shell.

Parameters	Values
**Data collection**	
Diamond Light Source beamline	I04-1
Wavelength (Å)	0.920
Detector	Pilatus 2M
Resolution range (Å)	32.64–1.95 (2.00–1.95)
Space group	P2_1_
Cell parameters (Å)	*a* = 41.43, *b* = 168.40, *c* = 133.90, β = 97.19°
Total no. of measured intensities	902,880 (59,341)
Unique reflections	131,515 (9597)
Multiplicity	6.9 (6.2)
Mean *I*/σ(*I*)	14.6 (1.6)
Completeness (%)	99.6 (98.6)
*R*_merge_*^a^*	0.092 (0.984)
*R*_meas_*^b^*	0.109 (1.190)
*CC*½*^c^*	0.998 (0.611)
Wilson *B* value (Å^2^)	22.1

**Refinement**	
Resolution range (Å)	32.64–1.95 (2.00–1.95)
Reflections: working/free*^d^*	124,837/6621
*R*_work_/*R*_free_*^e^*	0.197/0.238 (0.326/0.356)
Ramachandran plot: favored/allowed/disallowed*^f^* (%)	98.0/1.8/0.2
Root mean square bond distance deviation (Å)	0.012
Root mean square bond angle deviation (degrees)	1.47
No. of protein residues (ranges)	
A chain	446 (1–17, 23–451)
B chain	432 (1–17, 24–28, 41–450)
C chain	446 (1–17, 23–451)
D chain	446 (1–17, 23–451)
No. of water/MES/ethylene glycol molecules	652/4/4
Mean *B* factors: protein/waters/MES/ethylene glycol/overall (Å^2^)	35/34/33/30/35
PDB code	5LQD

*^a^ R*_merge_ = Σ*_hkl_* Σ*_i_*|*I_i_*(*hkl*) − 〈*I*(*hkl*)〉|/Σ*_hkl_* Σ*_i_I_i_*(*hkl*).

*^b^ R*_meas_ = Σ*_hkl_*(*N*/(*N* − 1))½ × Σ*_i_*|*I_i_*(*hkl*) − 〈*I*(*hkl*)〉|/Σ*_hkl_* Σ*_i_I_i_*(*hkl*), where *I_i_*(*hkl*) is the *i*th observation of reflection *hkl*, 〈*I*(*hkl*)〉 is the weighted average intensity for all observations *i* of reflection *hkl*, and *N* is the number of observations of reflection *hkl*.

*^c^ CC*½ is the correlation coefficient between symmetry equivalent intensities from random halves of the data set.

*^d^* The data set was split into “working” and “free” sets consisting of 95 and 5% of the data, respectively. The free set was not used for refinement.

*^e^* The *R*-factors *R*_work_ and *R*_free_ are calculated as follows: *R* = Σ(|*F*_obs_ − *F*_calc_|)/Σ|*F*_obs_|, where *F*_obs_ and *F*_calc_ are the observed and calculated structure factor amplitudes, respectively.

*^f^* As calculated using MolProbity (58).

**FIGURE 2.**
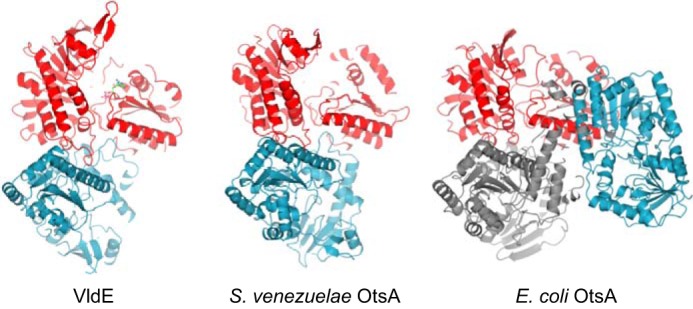
**The conservation of the *S. venezuelae* OtsA dimer interface.** The biological dimers of *S. venezuelae* OtsA, *E. coli* OtsA (PDB code 1UQT), and *S. hygroscopicus* VldE (PDB code 4F9F) (29) are shown with equivalent orientations with regard to the subunit depicted in *red*. The orientation of the second subunit within each dimer is depicted in *blue*, clearly showing a different orientation in the *E. coli* OtsA enzyme (21). However, the existence of a tetrameric form of the *E. coli* enzyme (PDB code 1GZ5; where the additional two subunits are shown in *gray*) shows that one subunit can be in the same orientation (20).

Four copies of the *S. venezuelae* OtsA protein were present in the asymmetric unit. Consistent with the size observed in solution, the biological unit appeared to be a dimer (Fig. 2). The two copies of the dimer within the asymmetric unit are essentially identical and had subunit interfaces of 1319 Å^2^ (30). The subunit interface in VldE (PDB code 4F9F) is topologically equivalent but more extensive at 2080 Å^2^. Interestingly, *E. coli* OtsA is also known to form a dimer in solution, but it is topologically different (PDB code 1UQT), giving a subunit interface of 1038 Å^2^ (21). However, it can also form a tetramer in solution (20) involving a second subunit interface of 1033 Å^2^ (PDB code 1GZ5) that is topologically equivalent to that of the *S. venezuelae* enzyme. Despite differences in quaternary structures, superposition of the A chain of *S. venezuelae* OtsA with *S. hygroscopicus* VldE (PDB code 4F9F over 368 residues) and *E. coli* OtsA (PDB code 1UQT over 393 residues) showed that they each had a common fold with a root mean square deviation value of 2.00 Å. The fold comprised twin Rossman-like β/α/β domains in a GT-B configuration. A molecule of MES buffer is bound in a cleft between the two domains of *S. venezuelae* OtsA in what is likely to be the active site, by analogy with the *E. coli* enzyme (20, 21).

**FIGURE 3.**
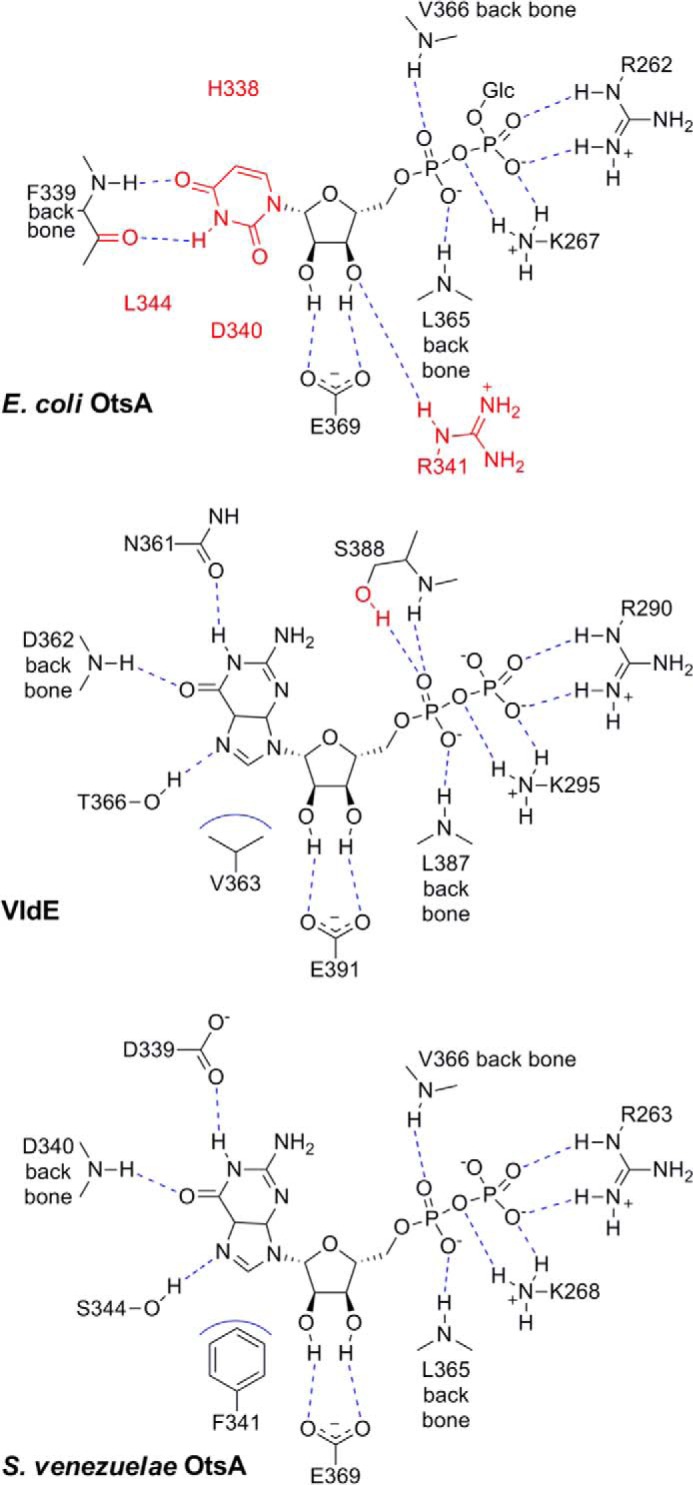
**The donor binding site is configured to bind GDP-glucose.** Two-dimensional maps show the binding interactions between UDP-glucose and *E. coli* OtsA (PDB code 1UQU) (21) and between GDP-glucose and *S. hygroscopicus* VldE (PDB code 4F96) (29). The interactions between GDP and *S. venezuelae* OtsA were predicted based on structural alignments with the known ligand-bound structures. Key differences between the three structures are highlighted in *red*, hydrogen bonds in *dashed blue lines*, and hydrophobic interactions in *solid blue curves*.

Attempts to obtain a structure of the *S. venezuelae* enzyme with either GDP or GDP-glucose bound were unsuccessful. Therefore, comparisons were made between the non-liganded structure and those of the closest structural homologues with nucleotides bound; *E. coli* OtsA with UDP-glucose bound (PDB code 1UQU) (21) and *S. hygroscopicus* VldE with GDP (and trehalose) bound (PDB code 4F96) (29). First, the active sites were superposed on the basis of the α-carbons of Arg-263, Leu-365, and Glu-369 (*S. venezuelae* numbering), which were conserved in all three proteins. Then, the observed and potential hydrogen-bonding interactions between the ligands and proteins were assessed (Fig. 3). It was immediately apparent that amino acid side chains associated with binding the ribose diphosphate were almost completely conserved. The only exceptions were an additional Arg-341 side chain interaction to the ribose in *E. coli* OtsA and an additional Ser-388 side chain interaction with phosphate in VldE.

With the exception of a hydrogen bond between a protein backbone NH and an oxygen of the base, the interactions with the base were quite different (Fig. 3). In the case of *E. coli* OtsA, there was an additional backbone CO interaction (from Phe-399) with an NH of the uridine base. By contrast, VldE formed hydrogen bonds between the side chains of Asn-361 and Thr-366 and the guanidine base together with a hydrophobic interaction involving a Val-363 side chain. All three of these interactions appear to be possible in *S. venezuelae* OtsA with its Asp-339, Ser-344, and Phe-341 side chains in equivalent positions to Asn-361, Thr-366, and Val-363 in VldE. Thus, the preference of *S. venezuelae* OtsA for GDP-glucose is evident from its structural similarity to VldE.

##### An S. venezuelae otsA Null Mutant Grows Like Wild Type on Maltose

Given the ability of *S. venezuelae* OtsA to utilize ADP-glucose to some extent as well as GDP-glucose, it was uncertain what the physiological donor would be, so we took a reverse genetic approach to address this. We had already established that blocking the GlgE pathway with a *treS* null mutation did not affect growth of *S. venezuelae* on complex or minimal media supplemented with maltose (8). This was because the product of TreS, maltose, bypassed the blockage (Fig. 1). Similarly, when the *otsA* gene was replaced by an apramycin resistance (*apr*) cassette, the resulting *otsA* null mutant was also able to grow on media supplemented with maltose, just like the wild-type strain. Metabolite analysis using NMR spectroscopy showed that trehalose levels were similar to the wild type (8) throughout growth on maltose. Although the maltose level reached 4% dry cell weight during growth, a little higher than the 2.5% observed in wild-type cells (8), it fell to zero during sporulation as normal. α-Glucan accumulated in pre-spore cells as normal, and spore morphology was like that of the wild-type strain, according to electron microscopy. No other changes in metabolite levels were apparent. Therefore, there the mutant strain did not show any significant phenotype on media supplemented with maltose.

##### The Mutant Strain Develops Slowly in a Cell Density-dependent Manner When Grown on Galactose

**FIGURE 4.**
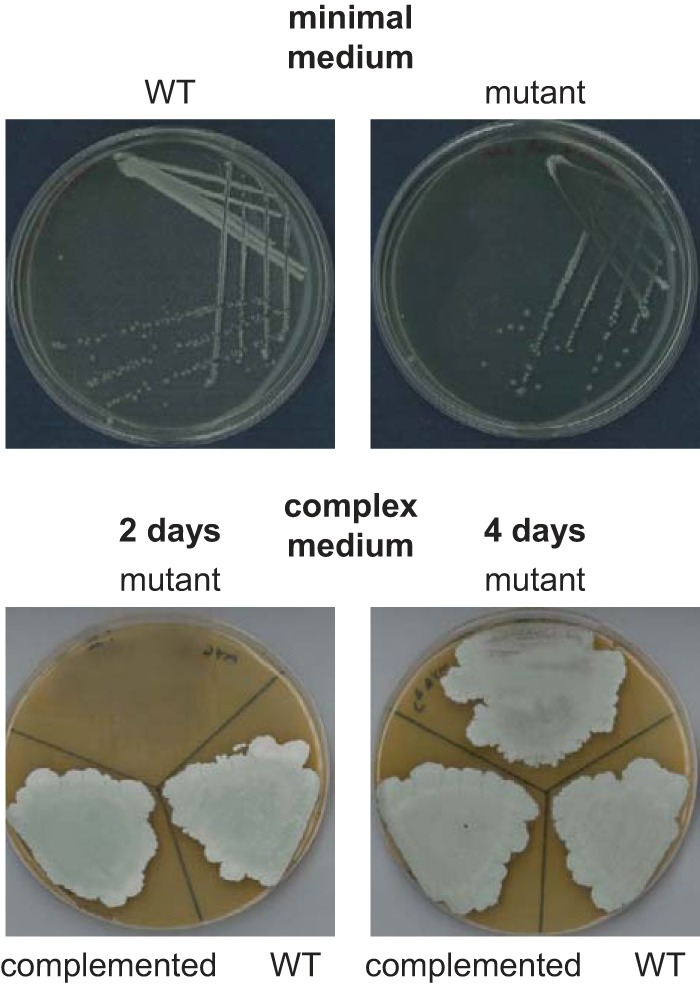
**Development of a *S. venezuelae otsA* null mutant is delayed in a density-dependent manner when grown on galactose.** The constructed Δ*otsA*::*apr* null mutant was grown on either minimal medium supplemented with galactose for 7 days or complex medium containing malt extract and supplemented with galactose for 2–4 days, as indicated. Growth and development were delayed in the mutant at high cell densities on both media. The complemented strain (Δ*otsA*::*apr attB*_Φ_*_BT1_*::*otsA*) grew like the WT strain.

We then tested whether a phenotype could be observed when the mutant was grown on a minimal medium supplemented with a carbon source other than glucose, maltose, or any other intermediate of the GlgE pathway. Although no growth phenotype of the mutant was observed when using fructose, there was a developmental delay with galactose (Fig. 4), such that sporulation did not appear to occur. Interestingly, the growth phenotype of the mutant was dependent on cell density, allowing isolated colonies to grow more like the wild-type strain.

To be able to isolate larger quantities of cells, the mutant was grown on a complex medium that contained both galactose and malt extract, the latter providing some maltose. A delayed growth phenotype was still observed, but sporulation did eventually occur (Fig. 4). With this growth medium, the mutant strain was able to generate some trehalose, most probably via TreY/TreZ or possibly via TreS (Fig. 1). However, the spores of the mutant strain contained less trehalose (5.4 ± 0.1% dry cell weight ± S.E., *n* = 3) than the wild-type strain (17.9 ± 0.4% dry cell weight ± S.E., *n* = 3). This suggested that the limited amount of maltose present in the medium was not sufficient to fully compensate for the lack of OtsA. Despite the lower level of trehalose, spore morphology was not affected, according to electron microscopy. Taken together, the mutant showed a delayed growth phenotype that was both galactose- and cell density-dependent.

##### The Mutant Strain Accumulates GDP-glucose When Grown on Galactose

**FIGURE 5.**
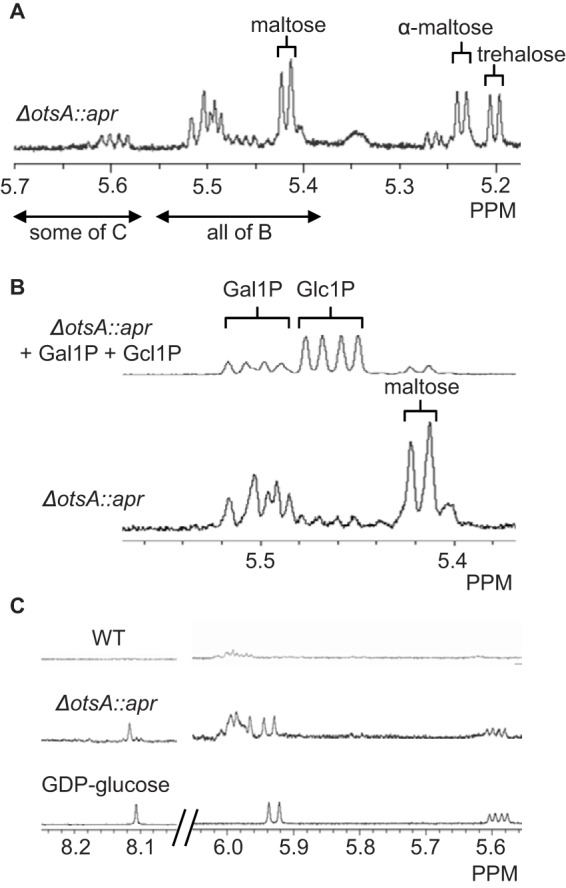
**The *S. venezuelae otsA* null mutant accumulates galactose 1-phosphate, glucose 1-phosphate, and GDP-glucose when grown in the presence of galactose.** The mutant (Δ*otsA*::*apr*) and WT strains were grown for 1.5 days on a complex medium supplemented with galactose, and cell-free extracts were analyzed using ^1^H NMR spectroscopy. *A*, the mutant accumulated trehalose, maltose, and possibly glucose. The regions of the spectrum overlapping with those shown in the subsequent panels are indicated. *B*, when the mutant extract was added to galactose 1-phosphate (*Gal1P*) and glucose 1-phosphate (*Glc1P*), the enhanced resonances lined up with those already observed in the extract (with different *y* axis scaling for clarity). *C*, additional resonances in the mutant extract compared with WT extract were consistent with the accumulation of GDP-glucose. A spectrum of authentic GDP-glucose is also shown.

^1^H NMR spectroscopy of cell-free extracts of the *otsA* mutant strain grown in the presence of galactose revealed the presence of a number of metabolites (Fig. 5*A*) that were also observed with the wild-type strain during growth (8). These included trehalose and maltose (and possibly glucose, given the overlap between the maltose and glucose resonances). There were, however, a number of other species that do not accumulate in the wild-type. For example, there were two sets of double doublets at 5.46 and 5.50 ppm (Fig. 5, *A* and *B*) that would be expected to arise from phosphosugars. Galactose is likely to be assimilated by *S. venezuelae* using a Leloir-type pathway in which galactose 1-phosphate is converted to glucose 1-phosphate via epimerization of an NDP-galactose (31). Indeed, adding the cell-free extract to authentic galactose 1-phosphate and glucose 1-phosphate enhanced these specific resonances, consistent with these two metabolites accumulating in the mutant.

In addition, another double doublet at 5.60 ppm (Fig. 5, *A* and *C*), together with additional resonances at low field, was suggestive of the presence of a nucleotide sugar diphosphate. Indeed, the resonances associated with authentic GDP-glucose were clearly present in the cell-free extract, consistent with the accumulation of this metabolite. The resonances of neither UDP-glucose, UDP-galactose, nor ADP-glucose were detected in the extract. Some additional broad resonances were also observed with the mutant extract, but the dominant ones are associated with glucose 1-phosphate, galactose 1-phosphate, and GDP-glucose. Therefore, GDP-glucose appears to be the donor of *S. venezuelae* OtsA *in vivo*.

##### S. venezuelae SVEN_2781 Can Generate GDP-glucose Efficiently

The bacterium *S. venezuelae* possesses several genes predicted to be NDP-sugar pyrophosphorylases, but it was not known which one(s) might produce GDP-glucose. With the absence of any known sequences of GDP-glucose pyrophosphorylases, we first identified three homologues of the GTP-dependent enzyme GDP-mannose pyrophosphorylase from *M. tuberculosis* (ManB Rv3264c) (32) in the genome of *S. venezuelae*. Then we produced these three enzymes as recombinant proteins in *E. coli*. First, SVEN_3027 is also a homologue of GalU from other organisms (also known as GtaB), which is normally associated with the formation of UDP-glucose. Enzyme assays that monitor the production of pyrophosphate showed that SVEN_3027 did possess NDP-glucose pyrophosphorylase activity such that the donor substrate preference was UTP > dTTP ≫ CTP > GTP > ATP > ITP. Therefore, this enzyme does indeed possess primarily GalU/GtaB-type UDP-glucose pyrophosphorylase activity and is not an efficient source of GDP-glucose.

Second, SVEN_3972 was isolated as a dimeric enzyme, according to size exclusion chromatography, that was able to produce GDP-glucose from glucose 1-phosphate (2 mm) plus GTP (1 mm). It seemed at first to be quite specific because it was inactive with glucose 1-phosphate plus any other donor tested (ATP, UTP, ITP, or CTP). However, it turned out to be most active with mannose 1-phosphate plus either GTP or ITP. The highest catalytic efficiency was with ITP and mannose 1-phosphate (Table 3). By contrast, the efficiency with which it generated GDP-glucose was over 100-fold lower, so its primary role seems to be the formation of IDP-mannose. Enzyme activity was not affected by the potential allosteric effectors glucose 6-phosphate, fructose 6-phosphate, GDP, GMP, or phosphoenolpyruvate up to 5 mm.

**TABLE 3:** **Kinetic analysis of *S. venezuelae* SVEN_3972**

Substrate	Fixed substrate (concentration)	*k*_cat_	*K_m_*	*k*_cat_/*K_m_*
		*s*^−*1*^	*mm*	*mm*^−*1*^ *s*^−*1*^
GTP	Glucose 1-phosphate (2 mm)	0.0055 ± 0.0004	2.5 ± 0.4	0.0022 ± 0.0004
Glucose 1-phosphate	GTP (2 mm)	0.014 ± 0.001	7.1 ± 0.8	0.0020 ± 0.0003
ITP	Mannose 1-phosphate (2 mm)	0.049 ± 0.001	0.17 ± 0.02	0.30 ± 0.03
Mannose 1-phosphate	ITP (1 mm)	0.0119 ± 0.0004	0.86 ± 0.08	0.014 ± 0.001
GTP	Mannose 1-phosphate (2 mm)	0.0156 ± 0.0004	0.47 ± 0.03	0.033 ± 0.002
Mannose 1-phosphate	GTP (1 mm)	0.0092 ± 0.0003	0.43 ± 0.05	0.021 ± 0.002

Third, SVEN_2781 did show relatively high activity with glucose 1-phosphate (1 mm) and GTP (0.25–1 mm) and was inactive with any other donor tested (ITP, ATP, CTP, or UTP). The production of GDP-glucose was confirmed using NMR spectroscopy. However, SVEN_2781 also exhibited a slightly higher activity with mannose 1-phosphate plus GTP (Table 4). This was perhaps to be expected because it shared 59% sequence identity with *M. tuberculosis* GDP-mannose pyrophosphorylase (32). NMR spectroscopy showed that with equimolar concentrations of donor and acceptor, the reaction went to completion. This showed that the chemical equilibrium was strongly in favor of the formation of GDP-mannose. The enzyme was present primarily in an active dimeric form, but inactive octameric and higher oligomeric forms were also detected using size exclusion chromatography. Enzyme activity was not affected by any of the potential allosteric effectors described above, in contrast to enzymes such as ADP-glucose pyrophosphorylase from other actinomycetes (27, 33).

**TABLE 4:** **Kinetic analysis of *S. venezuelae* SVEN_2781**

Substrate	Fixed substrate (concentration)	*k*_cat_	*K_m_*	*k*_cat_/*K_m_*
		*s*^−*1*^	*mm*	*mm*^−*1*^ *s*^−*1*^
GTP	Glucose 1-phosphate (1 mm)	0.159 ± 0.002	0.26 ± 0.02	0.61 ± 0.04
Glucose 1-phosphate	GTP (0.3 mm)	0.171 ± 0.004	1.4 ± 0.1	0.12 ± 0.01
GTP	Mannose 1-phosphate (2 mm)	0.168 ± 0.006	0.16 ± 0.03	1.1 ± 0.2
Mannose 1-phosphate	GTP (0.3 mm)	0.167 ± 0.008	2.7 ± 0.3	0.06 ± 0.01

## Discussion

We have now shown that *S. venezuelae* OtsA has a preference for GDP-glucose as the donor (Table 1), as has been reported for OtsA enzymes from other *Streptomyces* species (15–17) and other actinomycetes, such as *Rubrobacter xylanophilus* (18). Interestingly, not all examples of OtsA from actinomycetes share this donor specificity because the enzymes from mycobacteria show a preference for ADP-glucose (27, 34, 35). The best characterized OtsA enzyme to date has been that from *E. coli*, which has a preference for UDP-glucose. Several ligand-bound structures have allowed the residues that define its base specificity to be identified (Fig. 3) (20, 21, 36). Our structure of the enzyme from *S. venezuelae* shows that these defining residues are not conserved. By contrast, the equivalent residues that define the donor base specificity of VldE (29), which uses GDP-valienol, are similar in *S. venezuelae* OtsA. This helps explain why the *S. venezuelae* enzyme shows specificity for purine bases, with guanine being preferred. Interestingly, both open and closed conformations of *E. coli* OtsA and VldE have been described, where ligands tend to promote the open form (20, 21, 29, 36). Our structure of *S. venezuelae* OtsA most closely resembles the open conformations despite not having any ligands bound. In addition, there is a lack of electron density associated with a loop comprising amino acids 18–22 (peptide sequence GEDGE) of the *S. venezuelae* enzyme, implying disorder in this region. This loop is topologically in a similar position to a loop known to interact with the acceptor substrate in the *E. coli* enzyme that can be disordered in crystals lacking an acceptor bound to the active site (21). It is therefore conceivable that when ligands are bound, the *S. venezuelae* enzyme adopts a different conformation, and the GEDGE loop forms ordered contacts with the acceptor substrate.

An *otsA* null mutant of *S. venezuelae* exhibits no phenotype when grown on maltose because this GlgE pathway intermediate bypasses the need for OtsA to generate trehalose (Fig. 1). However, when grown on galactose, the mutant accumulates GDP-glucose (Fig. 5), showing that OtsA would normally utilize this donor substrate, although it is also capable of using ADP-glucose to some extent (Table 1). Then again, *S. venezuelae* does not possess an obvious *glgC* homologue that would code for an ADP-glucose pyrophosphorylase (5, 8), implying that this organism cannot produce ADP-glucose. The accumulation of both glucose 1-phosphate and galactose 1-phosphate is consistent with this organism assimilating galactose via a Leloir-type pathway (31). What was more unexpected was the cell density-dependent growth phenotype of the mutant when grown on galactose (Fig. 4). The accumulation of maltose 1-phosphate is known to lead to a delayed growth phenotype in *S. venezuelae*, but a lack of α-glucan has no effect (8). More dramatically, the accumulation of maltose 1-phosphate causes bacterial cell death in *M. tuberculosis* (4). It is therefore possible that the accumulation of glucose 1-phosphate and galactose 1-phosphate slows growth in *S. venezuelae.* Interestingly, the accumulation of ADP-glucose appears to be lethal in *M. tuberculosis* (37), so perhaps it is the accumulation of GDP-glucose that actually slows the growth of *S. venezuelae*. Either way, it is intriguing that the phenotype is cell density-dependent. Perhaps these or some other toxic metabolites are exported out of cells. Alternatively, perhaps some nutrients in the solid medium become locally limiting or metabolic fluxes are influenced by quorum sensing. Further studies will be required to establish the basis for the growth phenotype.

It is now clear that *S. venezuelae* OtsA utilizes GDP-glucose both *in vitro* and *in vivo*. We therefore sought an enzyme that is capable of generating this donor substrate. With the absence of a known sequence being associated with such an enzyme, we tested three candidate enzymes based on homology with the potentially similar GDP-mannose pyrophosphorylases. SVEN_3027 generated UDP-glucose as expected, given that it was also a homologue of GalU/GtaB from other organisms (33, 38). SVEN_3972, on the other hand, was somewhat unusual because its main activity appeared to be associated with the production of IDP-mannose (Table 3). Such an activity has rarely been reported (39). The values of *k*_cat_/*K_m_* with this enzyme were quite low (40). This implies that either the enzyme is inherently not very efficient or its primary role is to generate another nucleotide sugar diphosphate that has not yet been tested.

SVEN_2781 was able to generate GDP-glucose, even if it was also capable of forming GDP-mannose with slightly higher efficiency (Table 4). In contrast to SVEN_2781, GDP-mannose pyrophosphorylases from other organisms are specific for mannose-1-phosphate and do not utilize glucose-1-phosphate in all cases investigated (41, 42). The values of *k*_cat_/*K_m_* for SVEN_2781 were modest but well within the normal physiological range (40). Therefore, it seems reasonable to deduce that SVEN_2781 is capable of generating the donor substrate for OtsA *in vivo*. This enzyme therefore constitutes the first example of a GDP-glucose pyrophosphorylase (EC 2.7.7.34) of known sequence, although such enzyme activity has been documented several times (23–26). There remains the possibility that there are other pyrophosphorylases capable of generating GDP-glucose in *S. venezuelae*, and this can be explored in the future with reverse genetics and the characterization of the other pyrophosphorylases predicted to exist in this organism.

## Experimental Procedures

### 

#### 

##### Recombinant Proteins

Recombinant proteins were produced as described previously (4, 6). Genes were synthesized with optimum codon usage for expression in *E. coli* (Genscript Corp., Piscataway, NJ) to give proteins with tobacco etch virus protease-cleavable N-terminal His_6_ tags. *S. venezuelae* OtsA (gene locus synonyms SVEN15_3951 and SVEN_4043) was produced in *E. coli* SoluBL21. Cells were grown in lysogeny broth at 37 °C to an optical density of 0.6 at 600 nm before induction with 0.5 mm isopropyl β-d-thiogalactopyranoside (IPTG). After a further 20 h of incubation at 37 °C, cells were harvested by centrifugation for 10 min at 5000 × *g* at room temperature and disrupted by sonication (5 s on and 3 s off over 20 min on ice). The protein was purified using nickel affinity chromatography and stored in 5 mm HEPES, pH 7.0, containing 60 mm MgCl_2_. The size of OtsA was determined using size exclusion chromatography. A Superdex 200 10/300 GL column (GE Healthcare) was calibrated using blue dextran (2 MDa), β-amylase (200 kDa), alcohol dehydrogenase (150 kDa), bovine serum albumin (66 kDa), carbonic anhydrase (29 kDa), and cytochrome *c* (12 kDa), using 20 mm HEPES buffer, pH 7.0, containing 10 mm MgCl_2_ and 100 mm NaCl.

The pyrophosphorylases were produced in a similar way with modifications. SVEN_3027 (gene locus synonyms GalU, SVEN15_2964) was produced in *E. coli* BL21(DE3)pLysS, which was incubated at 28 °C after induction. The protein was stored at −80 °C in 20 mm Tris-HCl, pH 8.0, containing 100 mm NaCl and 10% (v/v) glycerol. SVEN_3972 (gene locus synonym SVEN15_3882) was produced in *E. coli* SoluBL21, which was incubated for 5 h after induction with 0.5 mm IPTG. After nickel affinity chromatography, the enzyme was applied to a Superdex 200 16/600 column equilibrated with 50 mm MOPS, pH 8.0, containing 0.025 mm EDTA and 5% (w/v) sucrose. Active fractions were concentrated, and 0.5 mm dithiothreitol was added for storage at −80 °C. SVEN_2781 (gene locus synonym SVEN15_2722) was produced in *E. coli* BL21(DE3), which was grown for 5 h at 18 °C after induction with 0.5 mm IPTG. The enzyme was stored at −80 °C in 50 mm Tris-HCl, pH 8.0, containing 0.5 mm dithiothreitol, 0.05 mm EDTA, and 5% (w/v) sucrose.

##### Enzyme Assays

Enzyme activity was quantified as described previously. Briefly, OtsA activity was determined by monitoring the production of NDP using a continuous coupled assay with phosphoenolpyruvate, pyruvate kinase, and lactate dehydrogenase, allowing the oxidation of NADH to be followed spectrophotometrically (27). NDP-sugar pyrophosphorylase activity was determined by monitoring the production of pyrophosphate using a stopped coupled assay with inorganic pyrophosphatase, allowing the production of inorganic phosphate to be detected spectrophotometrically with malachite green (43). Kinetic constants were calculated for each enzyme and condition using all replicate data using SigmaPlot version 13.0 with the Michaelis-Menten equation embedded in the Simple Ligand Binding macro. Compounds that could potentially allosterically regulate pyrophosphorylases were assessed at a concentration of 5 mm, and in each case, no impact on enzyme activity was detected. Enzyme-catalyzed reactions were also followed using ^1^H NMR spectroscopy. The consumption of the sugar 1-phosphate acceptors was monitored using their anomeric double doublet resonances at ∼5.5 ppm, relative to the signal from sodium 3-(trimethylsilyl)propionate-2,2,3,3-*d*_4_. The formation of the NDP-sugar products was monitored using their anomeric double doublet resonances, at ∼5.6 ppm, which were distinct from those of the donors.

##### Construction and Complementation of an S. venezuelae otsA Null Mutant

The bacterium *S. venezuelae* ATCC10712 was cultured at 28 °C in malt extract/yeast extract/maltose medium with 50% tap water (MYM-TAP) supplemented with 0.4 ml of trace element solution per liter (44). The maltose was substituted with other carbohydrates where indicated. The minimal medium (8, 44) was supplemented with 4–8 g/liter carbohydrate as a carbon source. The *S. venezuelae* null mutant in *otsA* (gene locus synonyms SVEN15_3951 and SVEN_4043) was generated using Redirect PCR targeting (45) to replace the coding region with an apramycin resistance (*apr*) cassette (8). The *otsA* gene was first replaced in the cosmid 1-H1,^4^ using the primers 5′-CGTTTGAGCGTTTACGGGACGGGCTAGGTTCGCCACATGATTCCGGGGATCCGTCGACC and 5′-CTGGAGCGGCCCCCACCTCGACAAGGTTCCAGGCGCTCATGTAGGCTGGAGCTGCTTC. The disruption of the cosmid was confirmed using restriction digestion with NruI. The cosmid was introduced into *S. venezuelae* by conjugation, and a double cross-over null mutant was selected on the basis of apramycin resistance and kanamycin sensitivity to give *S. venezuelae* Δ*otsA*::*apr* strain FM003. The deletion of chromosomal *otsA* was confirmed using Southern blotting with either XhoI or ApaLI. To complement the mutation, the *otsA* gene was amplified using 5′-GACCGGCCCAAGCCCACCC and 5′-TCAGGCGTCGCTCAGCCCC to give the open reading frame plus ∼300 base pairs upstream covering the native promotor. This fragment was cloned into pMS82 (46) to give pFM4, which was introduced into the mutant by conjugation to give *S. venezuelae* Δ*otsA*::*apr attB*_Φ_*_BT1_*::*otsA* strain FM003-pFM4 (FM013) where the plasmid is integrated at the ΦBT1 attachment site. This complemented strain had a wild-type phenotype. An empty vector control Δ*otsA*::*apr attB*_Φ_*_BT1_*::pMS82 strain (FM012) was also generated, which exhibited the phenotype of the mutant strain. The morphology of cells and spores was assessed using scanning electron microscopy, and the production of α-glucan was determined using transmission electron microscopy with periodic acid/thiocarbohydrazide/silver proteinate staining, as described previously (8). Spore stocks were standardized, allowing identical numbers of spores to be used to inoculate plates or cultures when assessing phenotypic differences.

##### Metabolite Analysis

Cells were grown on solid media overlaid with sterile cellophane discs and harvested by scraping (8). The cells were freeze-dried and powdered using a micropestle before being boiled, to denature enzymes, and disrupted by sonication. Cell debris was removed by centrifugation and cell-free extracts were analyzed by ^1^H NMR spectroscopy using sodium 3-(trimethylsilyl)propionate-2,2,3,3-*d*_4_ as an internal standard using assignments as previously described (6, 8).

##### Protein Crystallography

Protein crystallization trials were set up in 96-well MRC plates (Molecular Dimensions) using the following screens: JCSG-plus (Molecular Dimensions), PACT premier (Molecular Dimensions), Structure (Molecular Dimensions), Morpheus (Molecular Dimensions), ammonium sulfate (Qiagen), and PEG suite (Qiagen). Each reservoir was filled with 50 μl of the screen precipitant solution using the Freedom EVO liquid-handling robot (Tecan). A 0.3-μl sample of precipitant was mixed with 0.3 μl of protein (10–15 mg ml^−1^ in 5 mm HEPES, pH 7.0, containing 60 mm MgCl_2_) in a sitting drop format using an OryxNano robot (Douglas Instruments Ltd.). The plates were sealed and stored at 20 °C. Drops were monitored using a SMZ800 microscope (Nikon). After 3 days, rectangular plate crystals were present in the Morpheus condition 2-2 (0.12 m ethylene glycols (di-, tri-, tetra-, and penta-ethylene glycols), 0.1 m imidazole and MES, pH 6.5, 20% (v/v) ethylene glycol, and 10% (w/v) PEG 8000). Crystals were mounted directly from the screen in LithoLoops (Molecular Dimensions). They were then flash-cooled by plunging into liquid N_2_ and stored in Uni-Puck cassettes (MiTeGen) for transport to the Diamond Light Source (Oxfordshire, UK). Crystals were subsequently transferred robotically to the goniostat on the beamline and maintained at −173 °C with a Cryojet cryocooler (Oxford Instruments). Native diffraction data were recorded on beamline i04-1 (wavelength = 0.920 Å, 1800 images with 0.2° oscillation) using a Pilatus 2M detector (Dectris), processed using xia2 (47), and scaled using SCALA (48). A 5% subset of the total number of reflections was set aside to determine the free *R* factor (49) during model building and refinement. All subsequent processing was conducted using the CCP4 suite (50). The resultant data collection statistics are summarized in Table 2. The crystals belonged to space group P2_1_ with approximate cell parameters of *a* = 41.43, *b* = 168.40, *c* = 133.90 Å, β = 97.19°.

The structure of OtsA was solved by molecular replacement using programs from the CCP4 suite (50). The search model for molecular replacement was obtained by submitting the sequence to the Phyre^2^ server (28), which generated a template from the structure of *S. hygroscopicus* VldE (PDB code 3T5T), with which it shares 30% sequence identity. PHASER (51) was used to locate four copies of the protomer in the asymmetric unit giving an estimated solvent content of 46%. Density modification was carried out using PARROT (52), which benefitted from the use of 4-fold averaging and enabled the starting model to be entirely rebuilt with Buccaneer (53). Model building was then completed using COOT (54), alternating with refinement with REFMAC5 (55). The final model consisted of 1770 residues in four polypeptide chains, 652 water molecules, and four MES and four ethylene glycol molecules. The final *R*_work_ and *R*_free_ values were 0.197 and 0.238 to 1.95 Å resolution (refinement statistics are summarized in Table 2). MolProbity (56) was used to validate the model before deposition in the PDB. All structural figures were prepared using CCP4MG (57).

## Author Contributions

S. B. coordinated the study and wrote the paper. F. M., C. E. M. S., and D. M. L. carried out the crystallography. M. D. A. D. and A. A. I. designed the kinetic experiments and M. D. A. D. carried them out. F. M. carried out the microbiology. All authors analyzed the data, edited the manuscript, and approved the final version of the manuscript.

## Footnotes

4 M. J. Bibb and M. J. Buttner, unpublished data.

## Footnotes

3 The abbreviations used are:

PDB: Protein Data Bank
IPTG: isopropyl β-d-thiogalactopyranoside.
